# Neurophysiologic peculiarities of pediatric primary headaches

**DOI:** 10.1186/1129-2377-16-S1-A16

**Published:** 2015-09-28

**Authors:** Massimiliano Valeriani

**Affiliations:** Headache Centre, Ospedale Bambino Gesù, IRCCS, Rome, Italy; Center for Sensory-Motor Interaction, Aalborg University, Aalborg, Denmark

In spite of the high prevalence of primary headaches in pediatric age, most neurophysiologic studies in these diseases have concerned only adulthood. The neurophysiologic investigation of the pathophysiological mechanisms subtending migraine and tension-type headache in children and adolescents could be particularly interesting, since during the developmental age the migrainous phenotype is scarcely influenced by many environmental factors that can typically act on adult headache patients. Reduced habituation of evoked potential amplitude, that represents the neurophysiologic abnormality most frequently found in adult migraineurs, was confirmed also in migraine children, although it was shown to involve also children with tension-type headache. Some studies have shown abnormalities in the maturation of brain functions in migraine children and adolescents. While the visual system maturation is slowed in young migraineurs, the psychophysiological mechanisms subtending somatosensory spatial attention in migraine children are more similar to those of healthy adults than to those of age-matched controls. There are still some unexplored fields that will have to be subjects of future studies. In particular, the technique of transcranial magnetic stimulation, which has given an important contribution to our knowledge of primary headache pathophysiology in adults, has not yet been used in young migraineurs. It will possibly provide further elements about brain excitability in migraine children.

